# Review of Microgels for Enhanced Oil Recovery: Properties and Cases of Application

**DOI:** 10.3390/gels8020112

**Published:** 2022-02-11

**Authors:** Yulia A. Rozhkova, Denis A. Burin, Sergey V. Galkin, Hongbin Yang

**Affiliations:** 1 Faculty of Chemical Technologies, Industrial Ecology and Biotechnology, Perm National Research Polytechnic University, 614990 Perm, Russia; ketova.pstu@gmail.com (Y.A.R.); burinwork@gmail.com (D.A.B.); 2 Mining and Oil Faculty, Perm National Research Polytechnic University, 614990 Perm, Russia; 3 School of Petroleum Engineering, China University of Petroleum (East China), Qingdao 266580, China; yhb0810@126.com

**Keywords:** microgels, preformed particle gels, enhanced oil recovery, reservoir conformance control, pH-sensitive microgels, temperature sensitive microgels

## Abstract

In todays’ world, there is an increasing number of mature oil fields every year, a phenomenon that is leading to the development of more elegant enhanced oil recovery (EOR) technologies that are potentially effective for reservoir profile modification. The technology of conformance control using crosslinked microgels is one the newest trends that is gaining momentum every year. This is due to the simplicity of the treatment process and its management, as well as the guaranteed effect in the case of the correct well candidate selection. We identified the following varieties of microgels: microspheres, thermo- and pH-responsible microgels, thin fracture of preformed particle gels, colloidal dispersed gels. In this publication, we try to combine the available chemical aspects of microgel production with the practical features of their application at oil production facilities. The purpose of this publication is to gather available information about microgels (synthesis method, monomers) and to explore world experience in microgel application for enhanced oil recovery. This article will be of great benefit to specialists engaged in polymer technologies at the initial stage of microgel development.

## 1. Introduction

It has been estimated that an average of 210 million barrels of water and 75 million barrels of oil are produced worldwide every day. An excessive amount of water leads to undesirable consequences, including corrosion, scale formation, and a decrease in well efficiency [1]. The promising method for reducing the water cut of production oil well and decreasing residual oil saturation is the application of polyacrylamide gels.

Polymer gels are widely used in mature reservoirs due to their excellent profile control ability, easy preparation, and disproportionate permeability reduction property [2]. A new trend in gel preparation for EOR is the synthesis of preformed particle gels (PPG) having significant potential for conformance control [3]. PPGs do not have the disadvantages of other in-situ gels such as gelation time, insufficient strength in the presence of formation water [4], gel structure deformation due to shear degradation and changing of gelant composition induced by contact with reservoir minerals and fluids [5].

PPG is a macrogel with a particle size of more than 200 microns in dry form. These gels swell several times in water, and are injected directly into the well. Due to its viscoelastic properties, the gel’s particles are able to penetrate the high permeable layers [6]. However, the effectiveness of PPG macrogel use is limited by the high permeability of the reservoir, they are mainly effective at operational facilities with a permeability of 500 mD or more. To increase the applicability of PPG-based technologies in the oil industry, it is relevant to search for ways to use particles of a smaller granulometric composition. In this regard, a promising scientific field involves the development of reagents based on pre-cross-linked microgels having a particle size of up to 100 microns. From international experience, microgel application is effective in oil fields with a permeability starting from 10 mD. In this review article, we gather the available information on microgels developed and applied for EOR.

## 2. Methodology

In today’s world, due to machine learning [7] and other modern data processing technologies, multiple methods for search and systematization of relevant information for review research [8,9,10] now exist. One prospective approach is the PICO model. This method is used most commonly in health sciences, nursing, and medicine, but can also be adapted for application in other areas. The PICO recognition model consists of the following elements: P—problem, I—intervention, C—comparison, O—outcome [7,11]. It allows researchers to organize their work, break the topic into its key components, and make the article more precise. In our case, we will use the PICO model for research.

This article was organized based on the PICO technique. The first part of the article describes the problem of reservoirs that have been in operation for a long time. We consider this issue in connection with the problems experienced in Perm Krai, the territory of our research interest. The second part of the article is dedicated to microgels used for oil reservoir conformance control. We gather information on developed and in-development microgels. In the third part, we summarize the chemical approaches to microgel synthesis The article may be interesting for researchers at the initial stage of development of microgels for reservoir conformance control.

**Figure 1:**
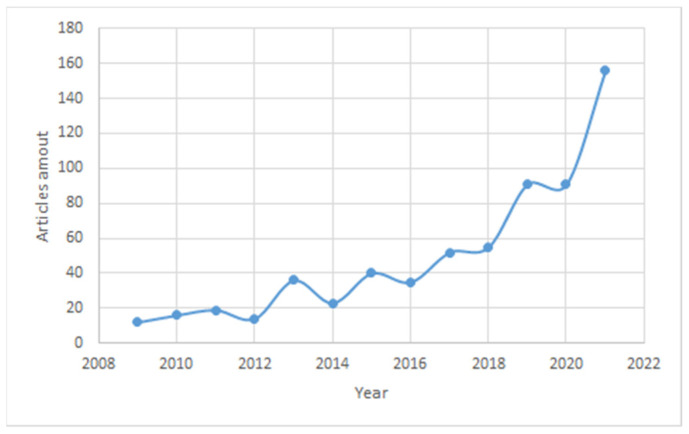
Increase in the number of publications since 2008 on the topic of microgels for enhanced oil recovery.

A search using the keywords “microgels for enhanced oil recovery” gives 942 articles published on the Science Direct website between 1998 and 2021. In the past year, the number of published articles has increased by more than 1.5 times, with 91 articles for 2020 and 154 for 2021. The graph in Figure 1 shows a constant increase in the number of articles, especially for the past year, which speaks to increasing interest in the topic of microgel application for EOR. The quality of work is guaranteed by the research data taken from the databases of Science Direct, ACS Publications, One Petro, etc. The main keywords used are “microgels”, “microgels for enhanced oil recovery”, and “preformed particle gels”.

### 2.1. Problem Description

The number of mature oil fields is constantly increasing [12,13]. One effective instrument for maintaining the production levels of these oil fields is by applying oil recovery enhancement methods, particularly reservoir conformance control methods [14,15]. One of the cheapest and environmentally friendly [16] reagents that is used for this is polyacrylamide (PAM), which costs about USD 2.00–4.00 per kg [17]. There are several approaches for PAM application in mature reservoirs: polymer flooding [18,19,20], bulk gels [21,22], preformed particle gels [17,23], and their combinations. The following comprehensive reviews are dedicated to polymer gels systems, including preformed particle gels and its application for enhanced oil recovery [17,24,25,26].

Preformed particle gels (PPG) were developed in 1996 by PetroChina [15,24]. PPGs are hydrogels synthesized by free-radical polymerization of acrylamide, cross-linker (usually N,N′-methylenebisacrylamide (BIS)), and other additives [27]. A PPG suspension prepared using any available water is injected into the reservoir. PPGs are able to swell up to 200 times in water [17]. The swelling particles of the hydrogel penetrate to the highly permeable fractured zones and block them. This helps redistribute injected water flows to the lowly-permeable oil-saturated interlayers of the reservoir. PPG technology allows reducing the water cut of the produced wells and increase the oil-well exploitation period.

PPG technology has been intensively developed in the last two decades. There are now more than 10,000 successful cases of its application [28]. It has allowed to overcome many difficulties and drawbacks of other PAM-application-based methods [25]. Experts developing this technology admit the following advantages:

- high selectivity: particles preferentially enter fractures and fracture-like channels and are unable to penetrate to low permeable oil saturated zones;
- simplicity of treatment: the suspension is usually prepared using only water (any available water with a wide salt concentration range is acceptable) and PPG; particles are easily dispersed in water;
- PPG properties: particles can be assigned their strength and size during synthesis on the surface; PPG particles have predictable properties in reservoir conditions due to their three-dimensional structure, while hydrogel particles are stable up to 120 °C [15,18,28,29].

**Figure 2:**
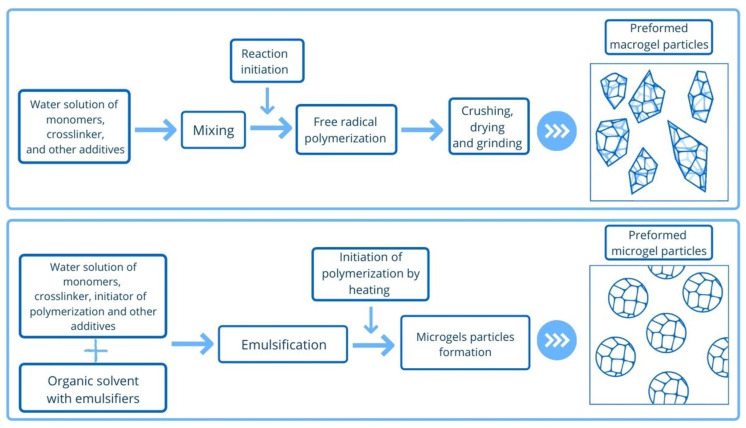
Types of preformed particle gels (macrogels and microgels) and principal schemes of their synthesis.

**Figure 3:**
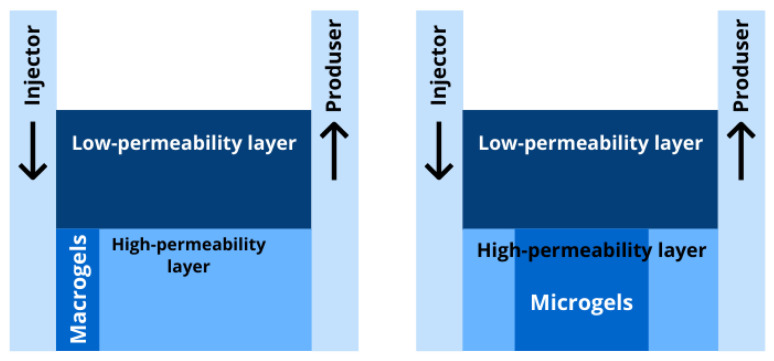
Zones of preferential reservoir conformance control by macrogels (**left**) and microgels (**right**).

Depending on size, PPGs can be divided into macrogels (more than 100 µm to mm) and microgels (less than 100 µm) [17]. The different approaches to microgel and macrogel synthesis are presented in Figure 2. Macrogels and microgels have different application conditions due to their difference in size. Macrogels are used for reservoir conformance control in the formation near the wellbore, while microgels are designed for the highly-permeable zones deep in the reservoir (see Figure 3).

It is known that the effectiveness of PPG macrogels is limited by the high permeability of the reservoir. As laboratory studies and pilot projects show, macrogels are predominantly effective at production facilities with a permeability of 500 mD or more [17,30]. To increase the applicability of PPG technologies in local oil fields, it is important to search for ways to use particles with a smaller particle size distribution. In this regard, a promising scientific area involves the development of microgels with a particle size of up to 100 microns. International experience shows that microgel application is effective in oil fields with a permeability starting from 10 mD [31].

The authors of this article are engaged in extensive research on PPG technology, particularly for application in the Volgo-Ural province. Oilfields have been under continuous development in the area for more than 50 years now. Extended oilfield exploitation leads to an increase in the water content in well production. We obtained a PPG adapted to the reservoir conditions of the Volga-Ural oil-and-gas province characterized by a low oil reservoir temperature (T < 30 °C) and a high mineralization of formation water (200–230 g/cm^3^). The PPG was synthesized in a concentrated solution of polyacrylamide with the addition of acrylic acid. During polymerization, the polymer chains cross-linked by imidization reactions between –COOH and –NH_2_ groups. PPG obtained using this method has a salt-water absorption capacity of 35–45 g depending on the salt concentration [32].

Analysis of the characteristics of 600 fields in the Perm Region, which can be considered a sample area for the Volga-Ural region, shows that only about 10% of production facilities have a permeability of more than 500 mD. With a decrease in permeability to 50 mD, the number of facilities sharply increases to 70% of the total fund. Therefore, the development of microgel compositions multiplies the number of potential facilities for the application. In this regard, a promising scientific field is the development of reagents based on pre-crosslinked microgels with a swollen particle size of less than 100 μm.

In this publication, we tried to combine the available chemical aspects of microgel production with the practical features of their application at oil production facilities. The purpose of this publication is to study the trends in microgel development (synthesis method, monomers) and explore world experience in microgel application for enhanced oil recovery. This article will be of great benefit to experts engaged in polymer technologies at the initial stage of PPG development.

### 2.2. Microgels for Reservoir Conformance Control

**Figure 4:**
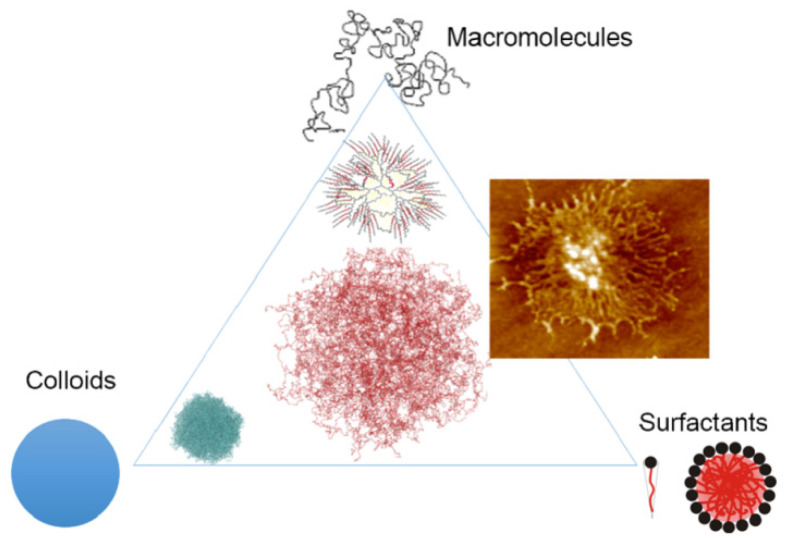
Schematic representation of microgels as systems with combined properties of three fundamental classes of colloids: flexible macromolecules, surfactants, and rigid colloids [35].

Microgels are particles of cross-linked polymers (gels) 0.1–100 μm in size (according to the IUPAC Gold book) having a three-dimensional structure and capable of swelling in a solvent [33]. The swelling process is caused by conformational changes of the cross-linked polymer network [34]. The interest in microgels is due to their unique properties, as they combine the properties of three groups of compounds: colloidal substances, polymers, and surfactants (see Figure 4) by compiling the unique properties of each class, including structural integrity, functionalization, softness, deformability, permeability, and others [35].

The most important characteristic of microgels is their average crosslink density that determines parameters such as the swelling ability (absorption capacity). Microgel swelling in an aqueous medium is determined by the balance between the solvent’s entropy, the energy of its interaction with the polymer chain, and the rigidity of the polymer chain [36]. The swelling capacity can also be improved by incorporating different functional groups into the microgel structure [37]. However, there are many factors affecting microgel absorption capacity, particularly temperature, ionic strength of the continuous phase, and pH. These factors can be used to control microgel size and depth of penetration into the reservoir. Several microgels with different properties are applied for EOR. In this part of the article, we will consider each of them.

#### 2.2.1. Colloidal Dispersed Gels (CDG)

CDG are microgels formed in-situ. Gel aggregates form in a lowly-concentrated solution of partially hydrolyzed PAM with a high molecular weight (more than 22 million Da) and a cross-linker (usually aluminum citrate of chrome citrate) [17,27]. The PAM concentration must be below the critical overlap concentration of the polymer, usually 100–1200 ppm. At this concentration, the polymer chains undergo intramolecular cross-linking, forming polymer coils. The ratio of the polymer to crosslinker concentrations vary from 20:1 to 80:1 [35]. The CDG globules formed may reach 1–150 nm in size [38]. The end of the CDG globules formation process is identified by a decrease in the solution’s viscosity [39]. CDG gels have been tested successfully in the fields of Argentina, China, and the United States [40,41,42]. An analysis of 31 cases of pilot tests is presented in the paper [43], the authors of which summarize the main parameters for implementing the technology on the well. The temperature of reservoirs where CDG was applied was 25–100 °C, reservoir permeability varied from 10 to 4200 µm^2^, and the oil viscosity of the treated deposits was 5–30 cPs.

During CDG injection, it is important to avoid any sudden pressure surges that could lead to gel transfer into the production well. The injection pressure can be controlled using the following parameters: injection rate, gel concentration, and polymer-to-crosslinker concentration ratio. In cases where the reservoir has the pronounced heterogeneity, CDG treatment is carried out after preliminary in-situ gel injection [44]. Depending on the injection pressure, CDG treatment can be changed in stages. In cases where the initial permeability of the formation is high, a high-concentration gel slurry is injected; after increasing the injection pressure, the PAM concentration is reduced [44].

CDGs are in-situ gels, i.e., microgel globule formation takes place inside the reservoir. The CDG technology requires low concentrations of polymer and crosslinker having inherent drawbacks of poor water production control. Moreover, factors such as shear degradation during injection, dilution by reservoir water, and interaction with minerals leads to a decrease in effective polymer concentration [45]. All these factors in a case of CDG application could lead to weak reservoir conformance control. The microgels considered further in this article have a significant difference in that they are synthesized before being injected into the dedicated equipment.

#### 2.2.2. Dispersed Particle Gel (DPG)

**Figure 5:**
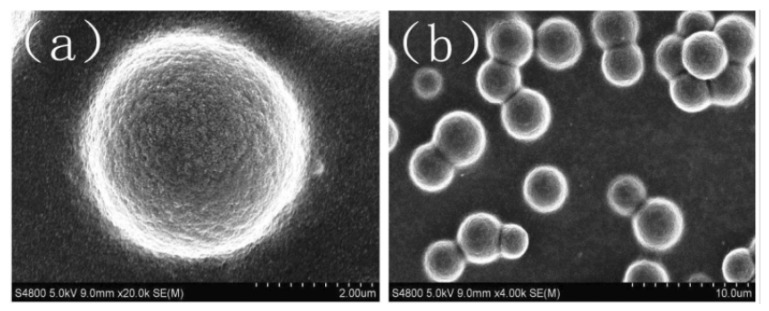
SEM images of dispersed particle gels (DPG) [48]. (**a**) spherical DPG particle, the size of particles may vary from 1.5 to 4 µm; (**b**) SEM photo demonstrate that DPG particles are able to form aggre-gates. The authors attributed that with the high surface energy of the particles.

DPGs are uniform spherical particles with the size adjusted from nm to mm (see Figure 5). The DPG receiving process occurs in two stages. The first is bulk gel formation. Here, gel strength and thermostability can be adjusted using the PAM with the suitable degree of hydrolyzation and a suitable cross-linker. The second stage is gel cutting by imposing high-speed shearing forces for several minutes [46]. A peristaltic pump [47] or colloid mill [46] can be used for the shearing. In the paper [46], research on DPG made of PAM and phenolic resin is described. The preparation procedure conditions are as follows: the first stage involves the formation of bulk gel at a temperature 75 °C. Thereafter, the gel is mixed with water (in similar proportions) and grinded using a colloid mill (3000 rpm, 3 min) to produce uniform particles 2.5 µm in size. In the article [38], the author used chromium acetate as a cross-linker. The results of experiments showed that Cr-DPG demonstrates good salinity resistance at 30 °C. The experiment for determining the thermostability showed that DPG size distribution dramatically changes after 15 days at 90 °C. The size of the lowest particles was halved (from 186.6 nm to 400 nm), and that of the highest particles increased by more than 5 times (from 796.2 nm to 4450 nm). Lab sand pack core flooding experiments on samples with a permeability ranging from 0.47 µm^2^ to 8.89 µm^2^ showed that DPG has good injectivity, which makes it an effective in-depth plugging agent.

In the article [49], a novel strengthened dispersed particle gel (SDPG) is presented. Silica nanoparticles (SiO_2_) were used as reinforced material to improve resistance to the high temperatures and high-salt content in the reservoir water. A non-ionic PAM of molecular weight 9,650,000 g/mol and a phenolic resin crosslinker were used. The SDPG obtained demonstrated their stability at 110 °C and a total salinity of about 213 g/L [50]. Several research describe the effective combination of DPG with surfactants [45,49,50]. The synergetic effect of the combination was confirmed during core flooding tests. The current research of the team of scientists who developed DPG focuses on self-growing hydrogel particles capable of growing after migration to deep fractures [51].

#### 2.2.3. Preformed Micro-Size Particle Gel

As mentioned above, PPG is a particle gel obtained from drying and grinding of the bulk gel [52]. These gels have a three-dimensional structure that forms during synthesis by cross-linking polymer chains with covalent polar bonds [30]. Preformed particle gels are polyacrylamide-based gels that absorb water and become soft and elastic. Their properties allow particles to penetrate into highly-permeable intervals of the formation. PPGs can be applied both on the fracture and on sandpack reservoirs [52,53,54]. Depending on their structures, PPGs have different mechanical properties that determine particle penetration into the permeable interlayers of rock. Weak PPGs have a better penetration capability and form a permeable crust on the surface of the lowly-permeable interlayer [55]. PPGs block the fractures partially because they are able to form channels for water passage. It is known that weak PPGs create internal channels more easily than strong gels [56]. Under harsh conditions (high salinity and temperature), ordinary PPGs shrink as a result of amide group hydrolysis and crosslinking by polyvalent metal from the water [57,58]. Nanocomposite PPGs with a superior stability and improved mechanical properties are presented in the paper [57]. The modified PPGs contained an equimolar ratio of the acrylamide, vinylpyrrolidone, 2-acrylamido-2-methylpropane sulfonic sodium salt; BIS as a crosslinker. The mechanical properties of the PPGs were enhanced by adding a dispersion of modified bentonite (MB) to the formulation. The nanocomposite PPS obtained demonstrated stability over 3 months at 130 °C. The swelling capacity in 25% total dissolved solids (TDS) solution was 9.53 g/g and that in fresh water 53.43 g/g [57].

The authors of the article [59] studied the matching factor of PPG in coreflooding experiments. Matching factor is the ratio of the PPG’s average diameter to the average pore-throat diameter. PPGs with a swelling particle average size of 9.1 µm were used in the experiments. Several cemented quartz cores were used with different permeability characteristics. The plugging behaviors of the PPG particles were summarized as three basic patterns: complete plugging (core permeability 26.26 mD); plugging-passing through in a deformation or broken state–deep migration (strong plugging in test on core with permeability 46.63 mD; general plugging in cores with 180.34 mD and 240.77 mD, weak plugging in core with 327.74 mD); inefficient plugging—smoothly passing through—stable flow (core permeability of 430.93, 633.29, and 857.86 mD).

Field trials of micro-sized PPG particles are represented in the paper [60]. Microgel size is described as, for example, 28 pm, which means the particles size is less than 28 µm. Particles were obtained from the grinding of bulk gel pieces. Microgel absorption capability at 125 °C in fresh water is about 23, and decreases to 6.5–7.0 when the brine TDS is more that 5%. Microgels are stable during at least one year at a temperature of 125 °C. For the trial treatment, a mature oil field in Northwestern China was chosen. The basic reservoir characteristics include a severe vertical and lateral heterogeneity and an average permeability of 230 md (max permeability was about 1500 md, water-cut of production wells was 95%). Treatment lasted 10 months, 169 tonnes of microgel were injected. The post-treatment effect lasted 18 months, and the quantity of additional oil was about 29.6 thousand tonnes, i.e., 175 tonnes of oil per 1 tonne microgel particles.

#### 2.2.4. SMG Microgels (Small Microgels)

SMG Microgels (Small Microgels) are a great example of acrylamide-based covalent cross-linked polymeric gels [31,61,62,63]. SMG are nontoxic [62]. Particle size varies between 0.3 and 2 µm. Their rigidity depends on the chemical composition, particularly of the cross-linker concentration. The three-dimensional structure gives the particles mechanical, thermal, and chemical stability. High shear-rate treatment (15,000 s^−1^) experiments have demonstrated stable viscosity for 16 min. SMGs are almost two times stable in brine containing H_2_S than in an ordinary PAM solution of a similar concentration. Thermal stability tests at 120 °C have demonstrated stable viscosity over three months. First, SMGs were considered for water shut off [64]; however, lab experiments on the SiC granular pack and natural sandstones demonstrated that SMGs have a great in-depth propagation. It has also been established that due to capillary forces, SMGs may be absorbed on pore walls forming thick layers, leading to water permeability reduction. The authors note that in these conditions, oil permeability remains unaffected. As conventional polymers, SMGs behave in the same way as relative permeability modifiers (RPM) [61]. The thickness of the absorbed layer can be regulated by varying the injected flow rate, microgel size, and concentration [31]. The paper [62] presents studies on how the high salinity of water affect SMG properties. These experiments showed satisfactory dissolution of microgels in brine with a salinity 215 g/L of TDS (sodium and calcium chloride). Layers adsorbing microgels tend to swell in low-salinity solutions and shrink when the salinity increases due to a charge screen on the gel particles surface. During coreflooding tests, the permeability reduction was fixed for a wide range of SMG suspension brine concentrations (20–108 g/L of TDS). When testing a suspension with a high salinity (200 g/L of sodium chloride and 15 g/L of calcium chloride), the post-treatment core permeability was found to be less. However, post-flush experiments allowed observing the hysteresis effect of microgel swelling behavior [62]. The first industrial case of successful SMG application was in 2005 for the treatment of an underground gas-reservoir storage [61,62]. In the paper [62], the authors present the first conformance control treatment using SMG. The chosen test well was an injection well surrounded by 7 producers. The basic reservoir had the following characteristic: permeability varying from 10–1000 mD (average around 200 mD), reservoir temperature 48 °C and reservoir water salinity 8000 ppm of TDS. A microgel suspension having a concentration of 500 ppm (1500 ppm of commercial solution) was injected over three months for a total volume of 9000 m^3^ (0.1 pore volume). Although the treatment pressure increased, it remained below the maximum authorized pressure. One year after treatment, the amount of additional oil was 1570 m^3^, and water production had been reduced by 23,830 m^3^ [31]. Two years later, the volume of additional oil was still increasing, reaching 5440 m^3^ after 26 months. 2.5 kg of microgel was injected for each tonne of additional oil [63].

#### 2.2.5. Microspheres

The authors of the paper [65] synthesized microgels by free-radical polymerization in inverse emulsion using diesel oil in a continuous phase. The chemical structure of the microspheres is formed by polyacrylamide cross-linked by BIS; a mixture of Tween-60 (polyethylene glycol sorbitan monostearate) and Span-80 (sorbitan monooleate) was used as an emulsifier. The absorption capability was determined by changing the particle diameter: the average diameter of the original macrogels was 50 nm, and after swelling, it reached several µm. The authors discovered that the emulsifiers used during the synthesis in combination with an additional surfactant or NaOH sharply reduce the oil/water interfacial tension during microsphere injection, leading to increasing residual and remaining oil saturation. Core flooding test on sandpacks models showed that microgel injection gives about 20% of additional oil. It was proved that microspheres have a great potential in EOR, particularly in reservoir profile control.

**Figure 6:**
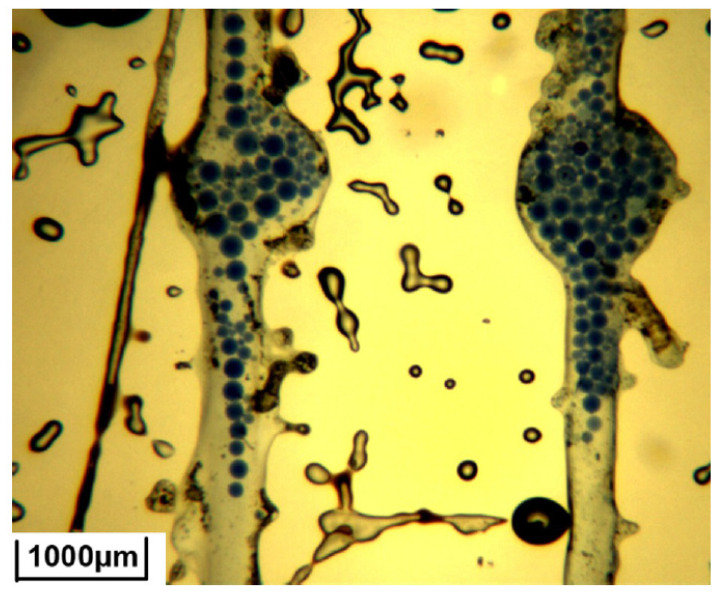
Distribution of microspheres in the micro-visual model [68].

Other examples of microspheres based on polyacrylamide and synthesized using the invers emulsion method are represented by different groups of researchers in the papers [66,67,68,69]. The BIS crosslinked elastic microspheres described in [66] swell in 3 days. The average microsphere diameter is 12.05 µm, the size of the swelling particle may increase to 25 µm depending on the temperature. In the salt solution (15–20 g/L), the size of particles is about 16–17 µm. Core flooding experiments on a sand pack demonstrated that the ideal matching factor (microsphere diameter/pore size ratio) is 1.35–1.55. At this ratio of microsphere diameter to pore size, gel particles are able to move while embedded deep in the sand pack. Authors of the article [68] presented a visualization of the process of pore filling by microspheres. A micro-visual model with a pore-throat size of 200–1000 µm was created. After microsphere dispersion pumping, some microspheres were accumulated and squeezed in the pore throat of the model, being used for plugging (see Figure 6). This observation proves that microgels are capable of changing the direction of the injected water to a reservoir’s oil-bearing interval [67].

Authors of the article [67] describe microsphere synthesis using the invers emulsion method; however, the difference is in the absence of any cross-linker in the co-monomers mixture (only acrylamide and acrylic acid). The average diameter of the microspheres obtained was 5 µm (Figure 5). After swelling, the particle size increased 5 times, which is more than with BIS cross-linked microgels described in the article [66]. Core flooding tests on the sand pack also proved the ability to redistribute injected water flows in the low permeable zones of the reservoir.

To adapt microsphere properties to the reservoir conditions, different types of microspheres with different viscoelastic properties were developed. The teams of authors of the papers [70,71,72] presented low elastic polymer microspheres given names such as L-EPM (i.e.) also synthesized using the invers emulsion method. The co-monomers of microspheres are acrylamide, acrylic acid, 2-acrylamido-2-metilpropansulfonic acid (AMPS), and BIS. Aviation kerosene was used as the continuous medium. The emulsifier was made from a mixture of Span-80 and Tween-60. The authors consider one important characteristic of microgels responsible for particle deformability and injection ability as storage modulus. The storage modulus G’ of L-EPM is 23.6 Pa. Experiments for determining microsphere behavior in the core pore space are presented in the paper [73]. For the test, sandstone cores with different porosities and pore sizes were taken. Depending on the microsphere-diameter-to-pore-size ratio, the following mechanisms of L-EPM penetration in the core were identified: (1) direct passing through the pore throat; (2) microsphere adhesion in the pores; (3) dehydration, stretching, extrusion, and retention to original form; (4) squeezing and breakage into pieces under pressure and its forward migration; (5) microspheres stack at the injection end of the sand pack, forming an external filter cake [48]. The coreflooding experiments showed that L-EPMs have a high selective profile control performance in remote heterogeneous reservoir zones [71].

Microsphere modifications are micron-size silica-reinforced polymer microspheres synthesized using the above-mentioned inverse suspension polymerization with the addition of 3-(methacrylyloxy)-propyl-trimethoxysilane (MPS) and silicon dioxide (nano sized) (authors call these microspheres such as PNSCMs) [74,75,76]. By adjusting the content of MPS-modified SiO_2_, the microsphere swelling ratio can be regulated well and the sensitivity of the swelling behavior to the environment is weakened. With increasing SiO_2_ loading, the microsphere mechanical stability, thermal stability, viscoelasticity, and dispersion stability were correspondingly improved [74]. PNSCM particle sizes vary between 10 and 100 µm. The swelling capability of SO_2_-modified microspheres was 35.5 g/g (for comparing the swelling capability of conventional microspheres was 47 g/g). Temperature has less effect on the swelling capability of silica-reinforced microspheres than that of conventional ones. This can be attributed to the introduction of modified SiO_2_. The maximum degradation temperature of silica-reinforced polymer microspheres was 430 °C (11 °C better than conventional microgels). In the article [76], the results of sand pack core flooding tests are represented. The main parameters of the experiment are as follows: the initial permeability of the core was 2.17 µm^2^, porosity about 30%, the resulting permeability decreases to 0.35 µm^2^. SO_2_-modified microspheres demonstrated excellent plugging properties in the micron-size pore throat, and the authors recommend it for deep conformance control application.

**Figure 7:**
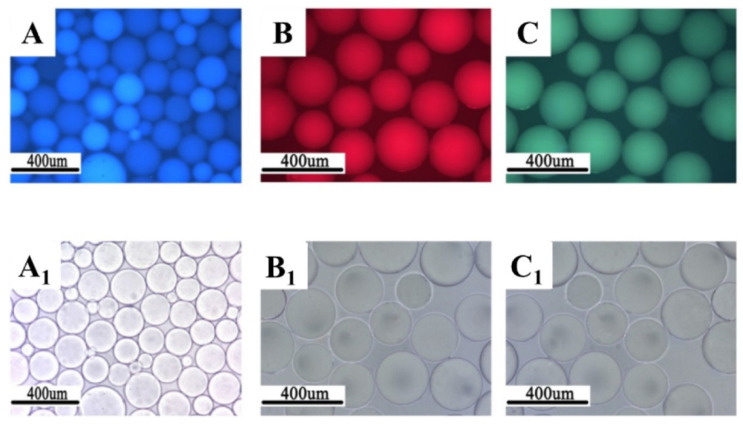
Morphology of fluorescent polymer microspheres (**A**) (co-monomer—cryloyloxy coumarin), (**B**) (co-monomer—allyl-rhodamine B), (**C**) (co-monomer—oxyfluorescein): under the uv light; (**A_1_**–**C_1_**): under the ordinary light) [77].

To detect microspheres in the reservoir-produced fluid, a new type of microspheres that fluoresce under ultraviolet irradiation was synthesized using an inverse suspension polymerization method [77,78,79,80]. The following fluorescent co-monomers were used for microgel synthesis: acryloyloxy coumarin [77,80], allyl-rhodamine B (RhB) [77,78], oxyfluorescein [77] (see Figure 7, Table 1). Since the concentration of fluorescent monomers was quite small, there were no significant changes in the initial particle size and the swelling property of the polymer microspheres.

#### 2.2.6. Thermal-Activated Microgels. Brightwater™ System (or “Popping” Microgel)

These microgels were developed by a consortium of BP, Chevron, Texaco, Mobil and Nalco Exxon Energy Services representatives. The idea of thermal-activated microgels is based on the fact that water injected into an oil reservoir is often cooler than the reservoir rock, which leads to formation of a temperature front somewhere between injection and the production wells [81]. Brightwater microgels are synthesized using the invers emulsion method in light mineral oil. The commercial form is 30% wt concentrate in light mineral oil. The chemical structure is based on highly cross-linked sulfonate-containing polyacrylamide. The three-dimensional structure is formed by two types of cross-linkers: stable and unstable. If the temperature rises the unstable cross-linker dissevers, particles absorb more surrounding water and expand. The author calls it the “popcorn effect”. Correct selection of cross-linkers provides particles with a sensitivity to the required temperature. Microgel particle sizes may vary from 0.1 to 3 µm. After popping, the particle size increases to 15–20 µm. Core fluid experiments on kernel particles have demonstrated that swelled microgels are able to penetrate the core with a permeability higher than 124 mD. The resistance factor and residual resistance factor values depend on the microgel concentration and core porosity [81]. Brightwater has been applied since 2001 starting from the Minas Field (Indonesia) [82]. There have also been trials in Brazil (Salema Field, Campos Basin) [83], and Alaska [84]. The main criteria for choosing the test oil-well for treatment include available movable oil reserves, early water breakthrough to high water-cut, porosity of highest permeable zones more that 17%, permeability in thief zones more than 100 mD, minimal reservoir fracturing, reservoir temperature 50–150 °C, and salinity of injection water not higher than 70,000 ppm [82]. The paper [84] presents tests of the Brightwater product in Alaska, Mylne Point field. The reservoir was generally homogeneous, with several macro-fractures mapped over the area. The tests were carried out on the area with three wells: one injection and two production wells. The production wells had a water cut of about 90%, the recovery factor was 20%. For processing, a microgel with a dry particle size of 0.1 to 1 μm was used, in swollen form—5 μm. Laboratory tests of the microgel demonstrated its stability for 2 years at elevated temperatures and a water salinity of 120 g/L. The reagent injection was divided into three sequential stages: (1) rapid injection of particles to pass the particles across the near wellbore formation zone; (2) filling the most permeable intervals with particles, heating to the formation temperature, destruction of temporary crosslinking; (3) popping and swelling of particles at a temperature of 50–75 °C. The temperature of the suspension when it acquires the perforation zone in the injection well was about 45 °C. The formation temperature at the production well in the same interval reached 80 °C. The treatment was carried out within 21 days. 60.8 tonnes of microgel suspension with a concentration of 3300 ppm were injected and 30.4 tonnes of surfactant were additionally pumped together. No change in injectivity was observed during the pumping. The first decrease in injectivity occurred 9 months after treatment. During the same period, recovery of additional oil was started for one oil production well. For another well, the production response was 11 months after treatment. The effect lasted 2 years. The total incremental oil volume was about 8000 m^3^ [84]. Experiments conducted on the test oil field demonstrated that Brigthwater is a thermally reactive particulate system that functions as an in-depth reservoir conformance control agent.

#### 2.2.7. pH-Activated Microgels

This type of microgel is highly sensitive to pH changes: the microgel suspension has low viscosity at low pH, with the viscosity increasing with an increase in the pH value. This gel is used for conformance control in remote formation zones: a microgel suspension is injected into a low-pH environment, small particles penetrate and move across the near-wellbore zone. During this process, acid from the microgel suspension reacts with rock minerals, leading to an increase in pH, microgels swell and block the highly-permeable zones deep in the reservoir. The pH-responsivity is provided by incorporation of co-monomers possessing carboxyl functional groups (e.g., acrylic acid) [85]. With increasing pH, the carboxylic groups of polyacrylate networks are hydrolyzed, and the charged groups repel each other. As a result, polymers chains are stretched and microgels swell [86]. To guarantee microgel penetration into the deep reservoir zone on the first stage of the reservoir treatment, pre-flush of formation by acids is recommended [86]. The experiments showed that acetic acid is better as a pre-flush agent [86,87]. The second stage is microgel injection, and the last stage of treatment is post water flooding. The higher water mineralization, the lower the pH value of weak acids, and the less swelling capability of microgels [87]. The most widely used pH-sensitive microgel is Carbopol^®^ (Lubrizol, Calvert, KY, USA). There have been several studies on the efficiency of microgels made using sandstone and carbonate cores [86,87,88,89,90,91]. The average size of Carbopol dry microgel particles is 2–7 μm. The permeability of cores used in the experiment was about 2.3 µm^2^ [87]. All experiments showed that at a pH of 2 (pH of microgel suspension), microgels block the injection end of the core. pH correction using NaOH helps overcome this problem [88]. During the flooding experiments, it was shown that these microgels efficiently plug the fractures and high permeable zones and redistribute filtration flows of water into the rock matrix. [86,87].

### 2.3. Microgel Synthesis Approaches

Several review articles have been published outlining different microgel production methods [89,90,91]. For microgels applied for EOR, the following basic approaches are used: balk gels synthesis and its mechanical grinding, precipitation polymerization, inverse emulsion polymerization.

The simplest microgel preparation method is mechanical grinding of bulk gel. Table 1 below describes two approaches. The resulting particle size differs at least 10 times. Therefore, the obtained product is aimed at different properties of the reservoir.

Another microgel synthesis method is precipitation polymerization. Three components are involved in the synthesis: monomer, crosslinker, and initiator [92]. The process is followed by a radical mechanism. At the polymerization temperature (50–70 °C), the water-soluble initiator (a compound based on peroxide or persulfates) decomposes on free radicals. In the case of persulfate, decomposition of the initiator leads to the formation of sulfate radicals that attack water-soluble monomers with subsequent propagation of radicals and chain growth [92]. When the microgel particles reach a critical size, they are stabilized by electrostatic stabilization mechanism. When microgel particles are formed, electrostatic repulsion prevents particles coagulation [93].

The precipitation method of monomer synthesis does not appear in publications on microgels for reservoir conformance control application. However, intra- and inter- molecular crosslinking of partially-hydrolyzed PAM polymer chains may be considered a special case of this method. An example of chemical crosslinking of polymer chains is CDG formation. Chemical cross linkers of partially-hydrolyzed PAM may be inorganic and organic in nature [94]. For CDG, aluminum citrate is the most common crosslinker. Chromium triacetate is used in fields with a high salinity of reservoir water. The polymer-to-crosslinker ratio may vary from 20:1 to 80:1 [95].

Crosslinking of PAM polymer chains may be achieved using not only chemical methods, but also a physical approach, particularly irradiation treatment. An example of crosslinking by radiation is a PPG-resembling technology used on Russian oil fields (Western Siberia and Tatarstan), and named similar to the polymer-gel system (PGS) Temposcreen [96]. This product is actually a macrogel, however, the approach could be considered for microgels synthesis in future. The three-dimensional structure of the polymer gel particles is formed by ionizing radiation at a dose of 10 kGy of PAM with a molecular weight of 20 × 10^6^ Da and a degree of hydrolysis of 30% [97]. The powder particle size distribution is 0.5–2 mm; in water, the particle can expand up to 1–10 mm in diameter. Depending on the size of the residual reserves and the geological structure of the reservoir, use of the Temposcreen PGM can yield 2–8 additional tonnes of oil. The technology was applied in high-temperature fields (85–95 °C) [97].

**Figure 8:**
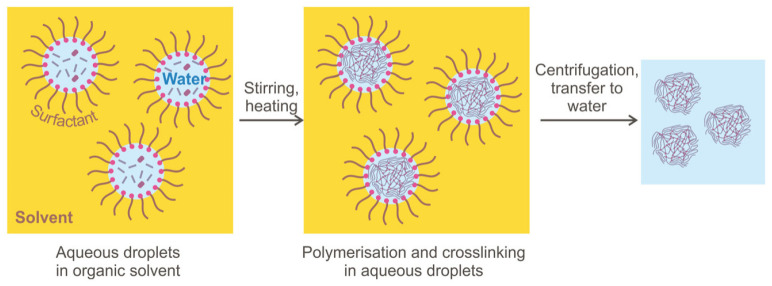
Microgel synthesis in water-in-oil emulsion [33].

The widespread microgel synthesis method for EOR application is inverse emulsion polymerization that has a different modification and allows obtaining different-sized particles (see Figure 8). Depending on the conditions (emulsifier concentration, stirring speed, initiator and dispersant concentrations), inverse emulsion-based polymerization may occur as suspension polymerization, microemulsion and nanoemulsion, giving microgels of different particle sizes. Although all types of emulsion are prepared using the same reagents (hydrocarbon solvent, water, and surfactant), the difference between these methods is the thermodynamic stability of both types of emulsions, which may influence the sizes of the microspheres obtained [90]. The inverse suspension method has many advantages: reaction heating control, granular product can be obtained without the grinding process; the product is easy to dry, and the resulting microgels have an excellent water absorption capability [80].

**Figure 9:**
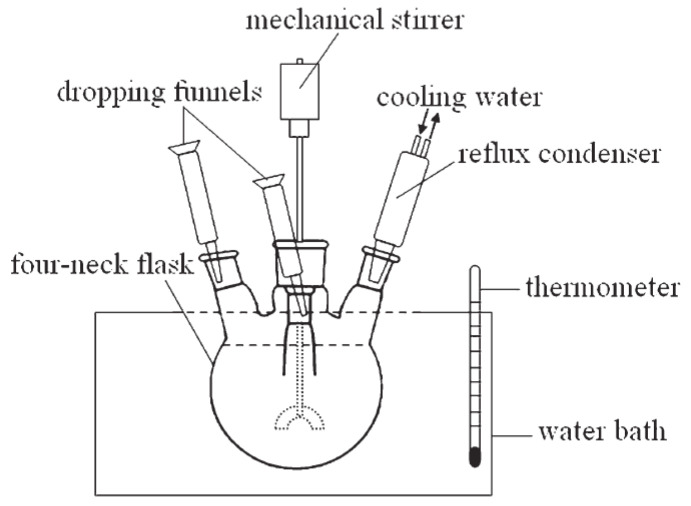
Diagram of laboratory unit for microgel synthesis using the invers emulsion method [78].

The main steps of the synthesis are: choosing the continuous phase (organic solvent, usually mineral oil or refined kerosene); selecting emulsifier systems; selecting a mixture of co-monomer and cross-linkers that will form physical and chemical properties of microgels; selecting an initiator system (for example, chemical initiation, then free radical are produced after chemical interaction, e.g., ammonium persulfate and sodium sulfite) [65,70,98,99]. The most important stage of inverse emulsion preparation is selection of the emulsifier mixture. The hydrophilic-lipophilic balance (HLB) of the emulsifier mixture must match the organic solvent HLB. Combinations of the following emulsifiers are usually used for inverse emulsion synthesis: Tween 60, Tween 80, Span 80, and other, [66,71,99]. As already mentioned above, such microgels such as microspheres and temperature- and pH-sensitive microgels have been synthesized using this method. The commercial products of microgels obtained using this approach is an organic solvent suspension (usually 30% mass). Figure 9 presents a diagram of the laboratory synthesis unit [66]. Table 1 lists compounds that are usually used for microgel synthesis using the invers emulsion approach. Examples of the synthesis of microspheres for reservoir conformance control are represented by the authors of the publications [66,67,68].

The microgel structure consists of the following fragments: polymer chains network, cross-linkers, and functionalized fragments embedded in the polymer backbone. Table 2 lists some compounds that are used for microgel synthesis and functionalization.

## 3. Conclusions

Several types of microgels have currently been developed. Table 3 summarizes the data about reservoir conditions where each type of microgel could be applied. Many laboratory and trial tests have demonstrated and proved the high efficiency of microgels for in-deep reservoir conformance control that is important today as the number of mature oil fields grows every year.

Incorporation of additional monomers into the microgels’ structure make it possible to obtain new unique characteristics: high strength [80,81,82], fluorescent [93,94,100], etc. Additional characteristics expand the microgels’ application ways. For example, fluorescent microgels can be used as markers to determine the formation lateral permeability.

Microgels have several advantages over other technologies for profile control with PAM application. These include high treatment selectivity, possibility of technology adaptation to reservoir conditions, easy treatment control, and guaranteed effect if the processing conditions are correctly followed. The current progress in microgel development demonstrates many possibilities for improving the technology relating to changing the mechanical properties (i.e., low elastic microspheres and SiO_2_-reinforced microgels) and incorporation of fluorescent monomers, which could improve lateral reservoir conformance control. Microgels can be considered the only component of injected suspension and in synergetic combination with surfactants and polymers solution that has a complex effect on the formation in terms of reducing residual and remaining oil saturation.

Based on the conducted review, the authors believe that technology of conformance control using microgels is a promising one having great prospects, especially in the development of mature oil fields.

## Figures and Tables

**Table 1:** Comparison of PPG and DPG synthesis.

Comparison Point	PPG	DPG
Synthesis feature	(1) synthesis from the monomers and crosslinker mixture; (2) initiation of free radical reaction of polymerization; (3) drying and cutting;	(1) gel formation from solution of partially hydrolyzed PAM and crosslinker; (2) heating (temperature depends on type of crosslinker); (3) mechanical cutting;
Commercial product	Dry powder	Suspension in water
Particle size	30 µm and higher	0.4–2.5 µm
Swelling process	Particles swell during suspension preparation of the oil field	Particles swell in the product

**Table 2:** Compounds used for microgels synthesis using the inverse emulsion method.

Compound	Formula	Function
Acrylamide	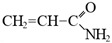	Scaffolding monomer
Acrylic acid	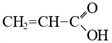	Scaffolding monomer, improving of hydrophilic properties
2-acrylamido-2-metilpropansulfonic acid	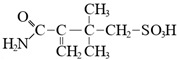	Scaffolding monomer, resistance to the high temperatures
N,N′-methylene-bis(acrylamide) BIS	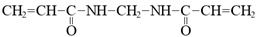	Cross-linking
3-(methacrylyloxy) propyl trimethoxysilane	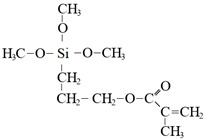	Reinforcing co-monomer for SiO_2_ encapsulation
Acryloyloxy coumarin	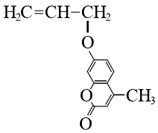	Fluorescent violet color
Allyl-Rhodamine B	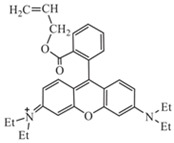	Fluorescent red color
Oxyfluorescein	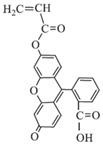	Fluorescent green color

**Table 3:** Microgels for EOR.

Type of Microgel	Synthesis Method	Particle Size	Target Reservoir Characteristics		
			Permeability	Water Salinity Limitation	Reservoir Temperature
Colloidal dispersed gels (CDG)	Precipitation polymerization	1–150 nm	10 to 4200 µm^2^	Depend on crosslinker type	25–100 °C
Dispersed particle gel (DPG)	Balk gels formation and its mechanical grinding	0.4–2.5 µm	0.47 µm^2^ to 8.89 µm^2^	213 g/L	stable under 110 °C
Preformed particle gel of micro size	Balk gels synthesis and its mechanical grinding	30 µm and higher	230–1500 md (average 230 µm^2^	wide range	stable under 125 °C
SMG Microgels (Small Microgels)	Inverse emulsion polymerization	0.3–2 µm	10–1000 µm^2^ (average around 200 µm^2^)	215 g/L	stable under 120 °C
Microspheres	Inverse suspension polymerization, inverse emulsion polymerization	About 12 µm and higher by inverse suspension polymerization and 0.3–2 µm by inverse emulsion polymerization	Wide range	wide range	stable under 120 °C
BrightwaterTM thermal-activated microgels	Inverse emulsion polymerization	0.1 to 3 µm	higher than 124 µm^2^	120 g/L	50–150 °C
pH-activated microgels	Inverse emulsion polymerization	2–7 µm	more than 10 µm^2^	Low salinity is preferable	-
